# Optimal Extent of Repair for Acute Type I Aortic Dissection—Frozen Elephant Trunk? How Long and Why?

**DOI:** 10.1055/s-0042-1756664

**Published:** 2022-12-15

**Authors:** Jessica G. Y. Luc, Ourania Preventza

**Affiliations:** 1 Division of Cardiovascular Surgery, Department of Surgery, University of British Columbia, Vancouver, British Columbia, Canada; 2 Division of Cardiothoracic Surgery, Michael E. DeBakey Department of Surgery, Baylor College of Medicine, Houston, Texas; 3 Department of Cardiovascular Surgery, Texas Heart Institute, Houston, Texas

**Keywords:** aorta, aortic dissection, stent graft, frozen elephant trunk, Type A dissection

## Abstract

Acute Type A dissection is a life-threatening condition requiring urgent surgical treatment. The operative technique involves repairs of a variety of distal extents of the transverse aortic arch and the downstream aorta. We review the evidence surrounding the extent of repair for acute Type A aortic dissection and describe our approach to this disease.

## Introduction

As described by the DeBakey classification system, type I dissection originates in the ascending aorta or root and propagates distally into the aortic arch and the descending and abdominal aorta, whereas type II dissection involves the ascending aorta or root and extends only to the origin of the innominate artery. 1 In the Stanford classification, Type A aortic dissection involves the ascending aorta or root and may extend into the arch and the descending and abdominal aorta. Thus, DeBakey types I and II dissection are both included in the Stanford Type A dissection classification. 1

According to the 2010 American College of Cardiology/American Heart Association guidelines, definitive surgical management of patients with acute type I aortic dissection is a class 1 recommendation to prevent morbidity and mortality. 2 The immediate goal of the operation is to eliminate the entry tear and to resolve any immediate malperfusion to keep the patient alive. The second goal would be to promote remodeling in the downstream aorta to prevent the need for subsequent procedures. Tube graft replacement of the ascending aorta from the sinotubular junction to the proximal arch (hemiarch) under circulatory arrest is the classic approach, based on the belief that decompressing the false lumen will result in the remodeling of the rest of the aorta. However, a standardized surgical approach for acute type I aortic dissection, regarding whether we need to address the distal aorta in the same operation as the proximal aorta, has yet to be defined. In this article, we review the evidence surrounding the optimal extent of repair for acute type I aortic dissection and offer our approach to this disease.

## Overview of Operative Approaches to Acute Type I Dissection

The majority of North American centers treat acute type I dissection by replacing the ascending aorta and proximal arch. Extended arch replacement is typically reserved for patients with arch or descending aortic dilation or tears involving the aortic arch. 1

An overview of the operative techniques and outcomes from North American centers for acute type I dissections has been previously published, with an overall mortality rate between 5 and 17%. 1 In recent reports, operative mortality was reportedly 15 to 18% and as high as 44% in patients with tamponade and 73 and 42% in patients with visceral malperfusion treated endovascularly and by surgical or hybrid procedures, respectively. 3 4 5 6 7 The following extents of surgical repair for type I aortic dissection have been described 1 :

- Ascending aorta and proximal arch replacement ± aortic valve/root repair or replacement.
- Ascending aorta and total arch replacement ± aortic valve/root repair or replacement.
- Ascending aorta and total arch/frozen elephant trunk replacement ± aortic valve/root repair or replacement.
- Ascending aorta and proximal arch replacement, with antegrade or retrograde stent delivery in the descending thoracic aorta by a thoracic endovascular aortic repair graft (TEVAR) ± aortic valve/root repair or replacement.
- Ascending aorta and total arch/elephant trunk replacement ± aortic valve/root repair or replacement.

The Canadian Cardiovascular Society/Canadian Society of Cardiac Surgeons/Canadian Society for Vascular Surgery Joint Position Statement on Open and Endovascular Surgery for Thoracic Aortic Disease 8 attempts to address the extent of distal repair for acute Type A aortic dissection. It makes a recommendation that an extended distal arch repair technique be considered for patients who present with acute Type A dissection and either a primary intimal entry tear in the arch or descending aorta or significant aneurysmal disease of the arch. 8 In addition, the writing committee suggested that it is reasonable to consider an extended distal arch repair technique for patients with acute Type A dissection and at least one of the following four characteristics: concomitant descending thoracic aortic aneurysm, distal malperfusion, young age, and known connective tissue disorder. In addition, the hybrid arch repair is to be considered for patients who are deemed too high risk for conventional open repair and who meet specific anatomic criteria. 8

The following questions remain to be answered in acute type I dissection:

- When should one do a proximal arch ± TEVAR versus a total arch ± frozen elephant trunk?
- When TEVAR is selected for the repair of the descending thoracic aorta, how long should the TEVAR stent be?
- When a frozen elephant trunk procedure is selected, how long should the frozen elephant trunk be?

## Proximal Arch versus Total Arch Replacement

In patients with acute type I dissection, the optimal surgical strategy remains controversial. Potential advantages of extended repair with total arch replacement rather than proximal arch replacement include a lower incidence of aortic-related complications in the downstream aorta and, consequently, less need for future reoperation. Risk factors previously identified for continued aneurysmal dilation of the descending aorta and subsequent need for reintervention include nonresected primary tear, patency of the false lumen, extent of dissection, and connective tissue disorder. 9 10 11 12 However, to experience the benefits of extended repair, the patient needs to survive the operation. Given the high surgical mortality associated with acute Type I aortic dissection, a more conservative, tear-oriented approach to aortic resection is generally favored. A branch-first total arch repair technique has been also described that shortens the distal organ and cardiac ischemic time and minimizes the morbidity associated with the repair. 13

A meta-analysis by Yan et al 14 of proximal arch versus extended repair with total arch and elephant trunk implantation in patients with acute Type A dissection showed that compared with extended repair, the proximal arch operation was associated with lower early mortality (risk ratio [RR] = 0.69, 95% confidence interval [CI]: 0.54–0.90, *p* = 0.005) but higher rates of postoperative aortic events, including reoperation of the distal aorta and significant dilation of the false lumen (RR = 3.14, 95% CI: 1.74–5.67, *p* < 0.001). Similarly, an early report 3 from the German Registry for Acute Aortic Dissection Type A study of type I dissection with entry site confined to the ascending aorta compared outcomes of patients who underwent proximal arch versus total arch replacement. This study found a tendency toward greater operative mortality for patients with total arch (25.7%) versus proximal arch (18.7%) repair; longer circulatory arrest times ( *p* < 0.001) were an independent predictor of 30-day mortality ( *p* = 0.041). These findings were consistent with those previously reported by Lio et al. 15

Rates of distal aortic reintervention at 5 years after proximal arch repair alone for type I dissection have been reported to be approximately 20 to 40%. 16 To examine the safety of reoperative elective open arch repair, if required, after type I dissection repair, we analyzed our contemporary experience with proximal arch versus total arch replacement over an 8.5-year period in patients with the previous repair of acute type I dissection. 17 Among 137 patients, 103 underwent a proximal arch replacement and 34 underwent a total arch or elephant trunk procedure. The operative mortality was 11.7%, the permanent stroke rate was 3.6%, and 5-year survival was 73.2%. These results suggest that elective reoperative arch replacement can be done with respectable overall morbidity and survival rates.

## Proximal Arch with or without Antegrade Stent Delivery

The first choice for intervention for complicated acute type III dissection is the use of endovascular grafts to cover the proximal entry tear, reduce pressure in the false lumen, treat malperfusion, and promote favorable aortic remodeling. 18 Using similar reasoning, we investigated whether delivering a covered stent (10-cm and 15-cm GORE TAG and Conformable TAG devices; W.L. Gore & Associates, Inc, Flagstaff, AZ) antegrade into the descending thoracic aorta during proximal arch repair for acute type I dissection affects the fate of the descending aorta. 18 Despite small numbers, we found that this treatment was safe, did not add to the circulatory arrest time, and was associated with the resolution of malperfusion. However, transient spinal cord ischemia (SCI) was more frequent in the stent group, albeit not significantly so.

**Fig. 1:**
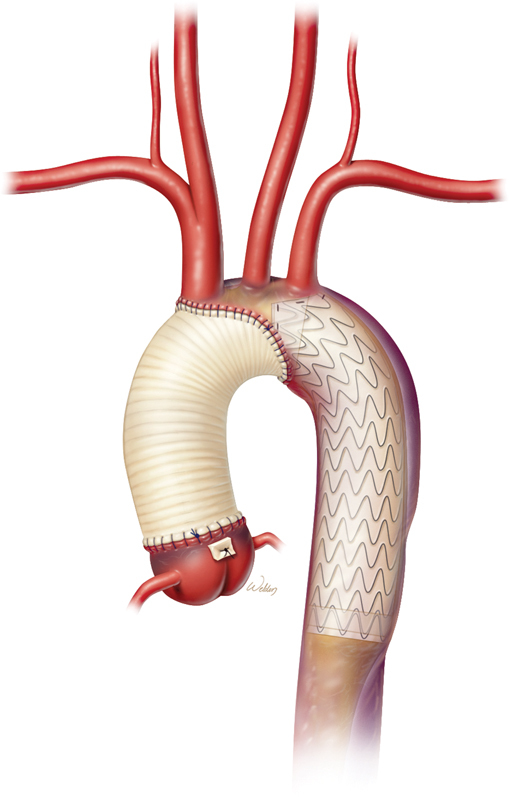
Ascending and proximal arch repair and antegrade stent delivery in a patient with acute type I aortic dissection.

In our follow-up study of mid-term outcomes in a propensity-matched series of patients who underwent either proximal arch replacement with stenting or proximal arch replacement alone for acute type I dissection, antegrade stent delivery was associated with greater remodeling but not with a significant difference in malperfusion resolution (stented 61.1% vs. no stent 72.2%, *p* = 1.00) or operative mortality (12.7 vs. 17.4%, *p* = 0.41; Fig. 1 ). 19 Rates of persistent paraplegia/paraparesis were similar in both groups (1.6 vs. 0.9%, *p* = 1.0), with the overall SCI rate being nonsignificantly higher in the stented group ( *p* = 0.18). With regard to the fate of the false lumen, computed tomography imaging (at 2.6 [0.7–3.8] years of follow-up for patients with stents and 4.0 [1.1–6.4] years of follow-up for the standard repair group) showed more complete thrombosis and remodeling in the stent group (63.5 vs. 29.0%, *p* = 0.002), with a trend toward fewer distal reoperations (9.3 vs. 16.0%, *p* = 0.250). Overall survival was better in the stented group at 3 years (73.3 ± 6.9% vs. 66.3 ± 9.4%) and 5 years (49.9 ± 7.6% vs. 41.6 ± 7.7%) ( *p* = 0.015 for both).

Consistent with our observations, Pochettino et al 20 found that in patients who underwent acute type I dissection repair, transient paraparesis was nonsignificantly more common in patients who had antegrade stent delivery to the descending thoracic aorta than in patients who did not (9 vs. 2%, *p* = 0.334). False-lumen obliteration was more frequent in the stented group, and this difference persisted at midterm follow-up (82 vs. 39%, *p* < 0.001). 21 The follow-up data also suggested that stenting promotes freedom from late open distal reoperation (98% at 6 years, vs. 90% in the nonstented patients, *p* = 0.01) by providing an endovascular platform in the thoracic aorta for future TEVAR if necessary. Other studies have also indicated that concomitant antegrade stenting during acute type I dissection repair is safe and promotes aortic remodeling. 22 23 Further investigation with longitudinal clinical and imaging follow-up is needed to determine the risks and benefits of proximal arch repair with antegrade stent delivery in patients with acute type I dissection.

In terms of stent graft length, our approach evolved with our experience to be more conservative with the stent length, limiting it to 10 cm instead of 15 cm to prevent coverage of T7 or T8, depending on the patient's body size, with 10% oversizing of the stent relative to the true lumen of the proximal descending thoracic aorta ( Fig. 1 ). The implantation technique has been previously published. 18 19 In brief, the stent is implanted during hypothermic circulatory arrest before the distal aortic anastomosis is constructed. We place a soft glide wire antegrade, under direct vision, down the true lumen of the descending thoracic aorta. We then exchange the soft wire for a stiff wire, over which we deploy the stent distal to the left subclavian artery. Circumferential 3–0 polypropylene sutures are placed to secure the stent by incorporating part of the stent graft into the distal suture line to prevent endoleak. Lately, we have started using the soft glide wire not antegrade but retrograde, coming from the femoral artery if feasible, to ensure deployment of the distal part of the stent in the true lumen.

## Total Arch Replacement with and without Frozen Elephant Trunk Procedure

In acute type I dissection, we opt for total arch replacement with or without a frozen elephant trunk procedure if the entry tear is within the greater curvature of the arch and cannot be repaired with a proximal arch operation, if there is a rupture or severe compression of the true lumen within the arch, or if there is dilatation of the arch (≥4.5–5.0 cm). Of note, proximal arch replacement with antegrade or retrograde stent delivery does not involve head-vessel reimplantation and, therefore, is not considered a frozen elephant trunk procedure. 18 When used for proximal acute aortic dissection, the frozen elephant trunk procedure promotes remodeling of the descending thoracic aorta and thrombosis of the false lumen better than the traditional elephant trunk procedure does. 24 25 The frozen elephant trunk may also facilitate a single-stage, rather than two-stage, replacement of the arch and proximal thoracic aorta, with open and endovascular options for subsequent extension of the repair. 24

We previously reported our lessons learned from experience in 129 patients with aneurysmal disease treated with either frozen elephant trunk or traditional elephant trunk procedures. 26 We found that compared with the traditional elephant trunk group, the frozen elephant trunk group had nonsignificantly higher rates of persistent spinal cord deficit and 30-day mortality. A position paper by the Vascular Domain of the European Association for Cardiothoracic Surgery 27 discusses the current status of and recommendations for the use of the frozen elephant trunk technique, concluding that its role remains to be defined and that when it is used, special caution is needed against the risk of SCI.

In our recent meta-analysis of frozen elephant trunk procedure outcomes in more than 3000 patients, 28 patients with acute Type A dissection did not differ significantly from patients with nonacute Type A dissection or aneurysm in the pooled mortality rate (9.2% [95% CI: 6.9–12.4%] vs. 7.6% [95% CI: 4.9–11.4%], *p* = 0.46) or stroke rate (9.3% [95% CI: 4.5–18.5] vs. 6.6% [95% CI: 3.1–13.5], *p* = 0.51). The pooled rate of SCI was lower among those with acute Type A dissection than among those with nonacute dissection or aneurysm (2.4% [95% CI: 1.3–4.2] vs. 5.2% [95% CI: 3.1–8.5], *p* = 0.05). However, the rate of overall composite adverse outcome, which comprised mortality, stroke, and SCI, was 22% for the patients with acute Type A aortic dissection versus 16.5% for the other patients ( *p* = 0.41). Notably, in this meta-analysis, the effect of SCI may have been masked by the trend toward higher mortality and stroke rates among patients with acute Type A dissection, which resulted in fewer of these patients being evaluated for spinal cord deficiency.

**Fig. 2:**
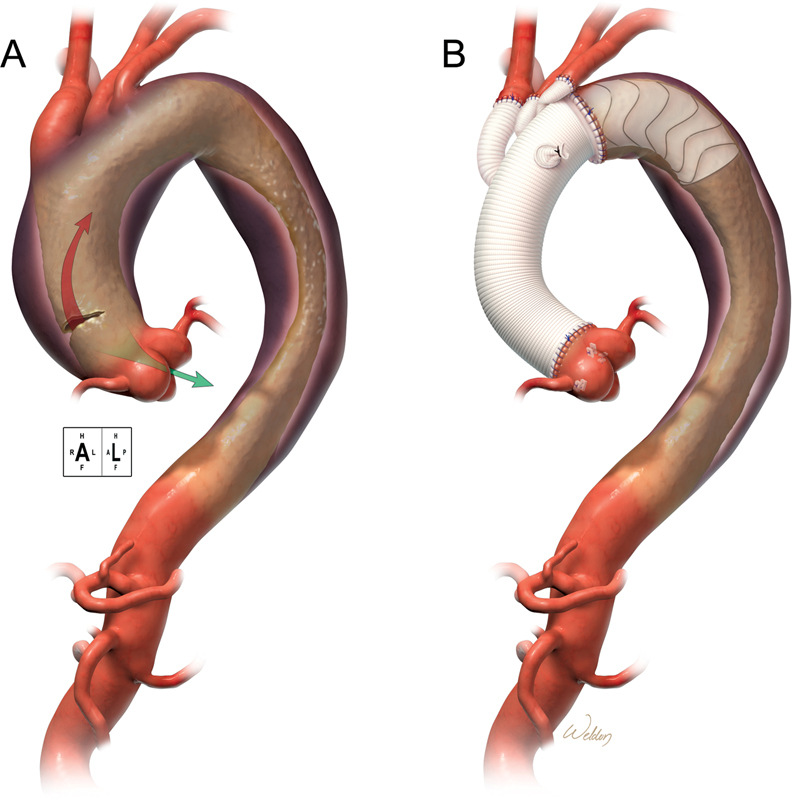
Total arch repair with frozen elephant trunk technique. ( **A** ) Acute type I aortic dissection. ( **B** ) Preferred stent length: 10 cm.

Regarding the effect of stent length on SCI rates, we showed that the overall SCI rate was 4.7% (95% CI: 3.5–6.2%) and that stent length >15 cm with coverage beyond T8 was associated with a significantly greater risk of SCI (11.6% [95% CI: 6.1–21.1] vs. 2.5% [95% CI: 1.5–4.0], *p* < 0.001). 28 We concluded that in the frozen elephant trunk procedure for acute type I dissection, a 10-cm stent is advisable ( Fig. 2 ) but should be used cautiously nonetheless.

Beyond stent graft length and patient size, other factors that may contribute to or protect against SCI have been suggested by Idhrees et al. 29 These include variations in spinal cord circulation, one- versus two-stage repair, lower-body protection during circulatory arrest, use of cerebrospinal fluid drainage, continuous perfusion of the left subclavian artery to provide collateral circulation to the spinal cord, atherosclerotic aortic plaque, deairing procedures, and postoperative management.

The Ascyrus Medical Dissection Stent (AMDS; Cryolife, Kennesaw, Georgia) hybrid prosthesis, which is a partially covered stent with a Teflon proximal cuff and uncovered Nitinol frame ranging from 15.5 to 22.5 cm in length, was used to repair acute type I dissection with acceptable midterm results in the recent single-arm Dissected Aorta Repair Through Stent Implantation trial. 30 The AMDS provides a reinforced distal anastomotic suture line that stabilizes the dissection flap within the aortic arch and descending aorta to promote favorable aortic remodeling. It also resolves malperfusion without adding too much complexity or morbidity to the procedure. The SCI rate was 0%, and the mortality rate was an acceptable 13% in a small cohort of patients. It remains to be seen whether AMDS stent implantation concurrent with acute type I dissection repair will reduce long-term reintervention rates; nonetheless, these findings suggest that the AMDS stent may be another adjunct that can be used in these patients with extended acute type I dissection repair.

## Our Approach

As we have advocated before, our preference is to keep things simple. Although our techniques have evolved over the years, our approach over the past 13 years for acute type I dissection has been to perform ascending aortic and proximal arch replacement (except in those cases, described above, in which total arch replacement is required). This is done without cross-clamping and under moderate hypothermia, with preferably bilateral instead of unilateral antegrade cerebral perfusion. In cases of malperfusion, we deploy a 10-cm stent antegrade into the descending thoracic aorta. We perform total arch replacement with frozen elephant trunk (the details of which have been published previously 31 ) and antegrade deployment of a 10-cm stent under direct vision in cases of entry tear within the greater curvature of the arch, rupture, or severe compression of the true lumen within the arch, and in cases of dilation of the arch (≥4.5–5 cm). If a stent is needed, then a 10-cm stent graft is deployed antegrade. Generally, a 10-cm stent is preferable to a 15-cm one because using a shorter stent can prevent SCI. 28

Proximally, aortic valve or root repair or replacement is performed, depending on the extent of the patient's disease.

## Conclusion

Acute type I dissection is a life-threatening condition requiring urgent surgical management. Operative techniques exist for acute type I dissection repairs with various distal extents. We suggest taking a patient-centered approach, tailored to the specific situation, the patient's condition, and extent of the disease. Caution should be taken with total arch replacement by the frozen elephant trunk technique because of the risk of neurologic complications.
